# Biotechnological Enhancement of Probiotics through Co-Cultivation with Algae: Future or a Trend?

**DOI:** 10.3390/md20020142

**Published:** 2022-02-15

**Authors:** Lucija Perković, Elvis Djedović, Tamara Vujović, Marija Baković, Tina Paradžik, Rozelindra Čož-Rakovac

**Affiliations:** 1 Laboratory for Aquaculture Biotechnology, Division of Materials Chemistry, Ruđer Bošković Institute, Bijenička cesta 54, 10000 Zagreb, Croatia; lucija.perkovic@irb.hr (L.P.); elvis.djedovic@irb.hr (E.D.); tamara.vujovic@irb.hr (T.V.); marija.bakovic@irb.hr (M.B.); rozelindra.coz.rakovac@irb.hr (R.Č.-R.); 2 Center of Excellence for Marine Bioprospecting (BioProCro), Ruđer Bošković Institute, Bijenička cesta 54, 10000 Zagreb, Croatia

**Keywords:** algae, probiotics, bioactive compounds, co-culture, nutraceuticals, human health

## Abstract

The diversity of algal species is a rich source of many different bioactive metabolites. The compounds extracted from algal biomass have various beneficial effects on health. Recently, co-culture systems between microalgae and bacteria have emerged as an interesting solution that can reduce the high contamination risk associated with axenic cultures and, consequently, increase biomass yield and synthesis of active compounds. Probiotic microorganisms also have numerous positive effects on various aspects of health and represent potent co-culture partners. Most studies consider algae as prebiotics that serve as enhancers of probiotics performance. However, the extreme diversity of algal organisms and their ability to produce a plethora of metabolites are leading to new experimental designs in which these organisms are cultivated together to derive maximum benefit from their synergistic interactions. The future success of these studies depends on the precise experimental design of these complex systems. In the last decade, the development of high-throughput approaches has enabled a deeper understanding of global changes in response to interspecies interactions. Several studies have shown that the addition of algae, along with probiotics, can influence the microbiota, and improve gut health and overall yield in fish, shrimp, and mussels aquaculture. In the future, such findings can be further explored and implemented for use as dietary supplements for humans.

## 1. Introduction

### 1.1. Algae as a Source of Bioactive Molecules for Human Well-Being

**Figure 1:**
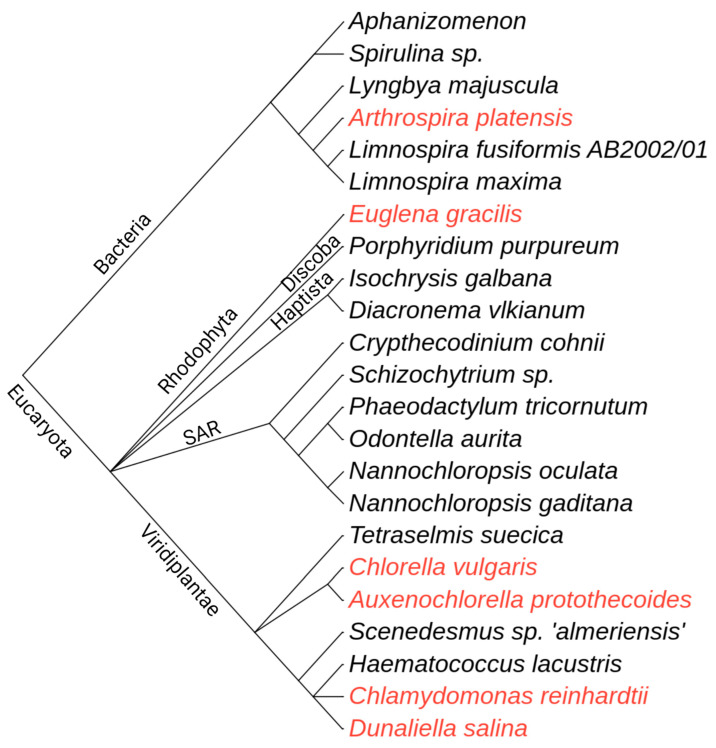
Phylogenetic relationships of microalgal species studied for their use in food or food ingredients. Species designated with red are approved as GRAS (Generally Recognized as Safe) by the FDA. The phylogenetic tree is built using the phyloT tool (https://phylot.biobyte.de/, accessed on 16 January 2022) and is based on NCBI taxonomy. The classification of *Shizochytrium* sp. as microalga is questionable [40].

Algae are predominantly autotrophic organisms that lack the true tissue organization of land plants. This large group of organisms includes prokaryotic organisms (Cyanobacteria) and phylogenetically diverse eucaryotic algae [1]. While microalgae are unicellular organisms, macroalgae (also known as seaweed) can grow to large scales—up to 50 m in length [2]. Both types of algae can produce biologically active metabolites and organic compounds [3,4]. Besides other biotechnological applications, these organisms have been frequently used in the food, feed, nutraceutical, and cosmeceuticals industries. Currently, the production of macroalgae is still dependent on wild stock harvest, with macroalgal aquaculture units increasing in recent years. Contrarily, photobioreactors are predominant production systems for microalgae [5]. Even though the estimated number of microalgal species is between 200,000 and 800,000 [6], only a few microalgae have GRAS (Generally Recognized as Safe) status as recognized by the FDA (Figure 1). In other countries, the number is even smaller and includes only a few species of *Chlorella*, *Arthrospira*, and *Dunaliella* genus. Contrarily, a larger number of microalgae species have been studied for their use as food or food ingredients [7]. These species belong to phylogenetically diverse organisms and represent a reservoir of various bioactive compounds (Table 1).

Many compounds (Table 1) isolated from microalgae show potential health benefits [8]. Among microalgae, *Chlorella* extracts have a wide range of different bioactivities, such as antioxidant, antibacterial, antidiabetic, antihypertensive, antihyperlipidemic, antitumor, and immunomodulatory effects, in humans and other mammals [8,9]. Commonly used as a dietary supplement, *Spirulina* has been found to be a source of γ-linolenic acid, phycocyanin, and high-quality protein. In addition, it possesses antiviral, anti-inflammatory, anticancer, and antioxidant properties. The health-promoting properties of pigments derived from *Spirulina* species show a major advantage in comparison to synthetic compounds [10]. In addition, several studies have demonstrated that *Spirulina* has neuroprotective properties and can support normal brain functions [11,12,13]. *Spirulina* extracts can reduce mental fatigue, prevent and/or mitigate cerebrovascular conditions, and ameliorate cognitive, motor, and language skills in malnourished children [14]. Finally, taking *Spirulina* supplements resulted in a significant reduction in all plasma lipids, total cholesterol, low-density lipoprotein, and triglycerides [15]. Another microalgae, *Haematococcus pluvialis*, is the richest source of astaxanthin, a commercially used carotenoid that has greater antioxidant activities compared to vitamin E and β-carotene [16]. The pigment β-carotene extracted from another algal species, *Dunaliella salina*, has been used in the food and cosmetic industries, as well as for the treatment of various medical conditions [17]. On the other hand, over 600 macroalgal species are categorized and used in food products [18,19]. Bioactive compounds extracted from some brown (Phaeophyceae), red (Rhodophyta), and green (Chlorophyta) algae have the potential of preventing and treating neurodegenerative diseases [20]. Monoterpenes extracted from macroalgae show anticancer, antiplasmodial, and insecticidal activity [21]. In addition, phytosterols, especially fucosterol, also seem to have health benefits, including anticancer, antidiabetic, immunomodulatory, anti-obesity, anti-inflammatory, neuroprotective, and many others [22]. Finally, pigments derived from various macroalgae are shown to have antioxidant activities using in vitro and in vivo assays [23,24,25].

Food products supplemented with compounds extracted from algal biomass have been shown to have various positive impacts on animal and human health. For example, the addition of *Chlamydomonas reinhardtii* biomass to the diet has a positive impact on gastrointestinal function in mice and humans. In mice, the addition of *C. reinhardtii* biomass mitigated weight loss, while, in humans, *C. reinhardtii* reduced the occurrence of gastrointestinal symptoms, such as bowel discomfort, bloating, diarrhea and gas, and increased stool quality [26]. The consumption of supplements of macroalgal origin that contain certain polysaccharides and phlorotannins may also have health-promoting effects on the digestive tract, as well as in the prevention of diabetes, osteoporosis, cancer, and cardiovascular diseases [27]. Apart from the food industry, the demand for extracts, microalgal and macroalgal biomasses, as well as natural and environmentally sustainable cosmetic products, has also been increasing because of their antioxidant, antiaging, moisturizing, and UV-screening properties [28,29,30]. *Chlorella* and *Arthrospira* are the most used microalgae in the skincare industry. *Arthrospira* extracts are efficient in correcting early signs of skin aging and preventing the formation of stria, while *C. vulgaris* extracts stimulate collagen synthesis and, consequently, cause wrinkle reduction [31]. Carotenoids (e.g., β-carotene, astaxanthin, fucoxanthin) have been used in beauty products, such as lotions and creams, as biologically active ingredients with antioxidant and nutritional value [32,33]. Carotenoids from *D. salina* and *H. pluvialis* have been used as colorants and protection from UV rays [34]. In line with this, carotenoids extracted from *Tetraselmis* spp. have a positive impact on epidermal tissue growth, as well as reducing hyperpigmentation, the size of melanocytes, and skin tension [35]. Different metabolites (polysaccharides, fatty acids, proteins, phenolic compounds, pigments, sterols, etc.) derived from macroalgae have also been used in cosmetic products. However, the most abundant and beneficial are polysaccharides (fucoidans, laminarins, alginates, etc.) because of their therapeutic applications [2,36].

Generally, the diversity of algal species is a rich mine of many different bioactive metabolites (Table 1). Significant efforts have been made in the past to obtain algal axenic cultures in order to increase the production of biomass. However, co-culture systems are emerging as an interesting solution that can tackle the high risk of contamination in axenic cultures, therefore increasing productivity and synthesis of active compounds [37]. In fact, algae are known to be a part of the phytobiome (plant microbiome), where they exhibit antimicrobial effects and have beneficial effects on plants [38]. Interactions between microalgae and bacteria have shown great potential in recent years to improve algal biomass production and enrichment of its composition with compounds of industrial interest [39]. In this review, we will encompass the research exploring the mutual improvement between algae and different microorganisms using co-culture systems. Since probiotic organisms represent an interesting partner for the usage in food and cosmetics, the main focus will be on recent research that combines these two types of organisms in order to implement their metabolic products in nutraceuticals.

**Table 1:** Overview of some microalgal species studied for food and food ingredients, and their main bioactive molecules. Species marked in red are approved as GRAS by the FDA.

Microalgae	Fatty Acids	Pigments	Other	Ref.
*Spirulina* sp.	/	/	Polysaccharides	[41]
*Aphanizomenon flos-aquae*	/	/	Mycosporine-like amino acids (MAA)	[42]
*Lyngbya majuscula*	/	/	Lyngbic acid, malyngamides, grenadadiene, debromogrenadiene, grenadamide	[43,44]
*Arthrospira platensis*	Monounsaturated (oleic acid) and polyunsaturated (γ-linolenic acid, DHA)	Zea, Ast, β-Car, Lut, Cantha	/	[45,46,47]
*Limnospira maxima*	Polyunsaturated (γ-linolenic acid)	C-PC, β-Car	α-tocopherol	[48,49,50]
*Limnospira fusiformis*	/	C-PC, β-Car	α-tocopherol, α-lipoic acid	[51,52]
*Porphyridium purpureum*	Saturated (palmitic acid), monounsaturated (palmitoleic acid), and polyunsaturated (EPA, arachidonic acid)	β-Car, Chl *a*, Zea, Chlide *a*, Cry, Phe *a*, Pheide *a*, PE, PBPs	/	[53,54,55]
*Euglena gracilis*	Polyunsaturated (EPA and DHA)	β-Car, Zea, Diato, Diadino, Neo	Paramylon, α-tocopherol	[56,57,58]
*Isochrysis galbana*	Polyunsaturated (EPA and DHA)	Fuco, Chl *a*	Amino acids (Arg, Met, Lys, Thr, Phe, His, Ile, Leu, Val, Trp)	[59,60,61]
*Diacronema vlkianum*	Saturated (myristic and palmitic acid), monounsaturated (palmitoleic acid), and polyunsaturated (stearidonic acid, EPA, and DHA)	Fuco, Lut, Zea, β-Car, Chl *a*, Chl *c*, Ast	α-tocopherol, p-sitosterol, stigmasterol	[62,63,64]
*Crypthecodinium cohnii*	Polyunsaturated (DHA)	/	/	[65]
*Schizochytrium* sp. *	Polyunsaturated (DHA)	Ast, Cantha, β-Car, Ech	/	[66,67]
*Nannochloropsis gaditana*	Polyunsaturated (EPA)	Ast, Cantha, Chl *a*	/	[68,69,70]
*Nannochloropsis oculata*	Polyunsaturated (EPA)	/	α-tocopherol	[71,72]
*Odontella aurita*	Polyunsaturated (EPA)	Chl *a*, Fuco	/	[73,74]
*Phaeodactylum tricornutum*	Polyunsaturated (EPA)	/	/	[75]
*Tetraselmis suecica*	Polyunsaturated (EPA)	Chl *a*, Chl *b*, α-Car, γ-Car, Lut, Lo, Viola, Neo, Ax	/	[76,77]
*Auxenochlorella protothecoides*	Saturated (palmitic and stearic acid), monounsaturated (oleic acid), and polyunsaturated (linoleic and linolenic acid)	Lut	/	[78,79]
*Chlorella vulgaris*	Saturated (palmitic and stearic acid), monounsaturated (oleic acid), and polyunsaturated (linolenic acid)	Ast, β-Car, Lut, Cantha, Lyco	/	[80,81]
*Scenedesmus* sp. *“almeriensis”*	Saturated (stearic, palmitic, and lauric acid), monounsaturated (oleic acid), polyunsaturated (linoleic and α-linoleic acid)	Lut, Ast, β-Car, Chl *a*, *b*, *c*	Haemagglutinin, MAA, amino acids (Ile, Leu, Met, Lys, Ala, Val, Arg, Cys and others), vitamin B, C, E	[42,82]
*Haematococcus lacustris*	/	Ast	/	[83]
*Chlamydomonas reinhardtii*	Saturated (palmitic acid), monounsaturated (oleic acid), polyunsaturated (α-linoleic and linoleic acid)	Chl *a*, *b*, Lut, β-Car,	/	[84,85,86]
*Dunaliella salina*	/	β-Car, α-Car, Zea, Lut	Sterols (7-dehydroporiferasterol, ergosterol)	[87,88]

MMA—mycosporine-like amino acids; α-Car—α-carotene; β-Car—β-carotene; γ-Car—γ-carotene; Zea—zeaxanthin; Ast—astaxanthin; Lut—lutein; Cantha –canthaxanthin; Fuco—fucoxanthin; Chl *a*—chlorophyll a; Chl *b*—chlorophyll b; Chl *c*—chlorophyll c; Chlide a—chlorophyllide a; Cry—cryptoxanthin; Phe a—pheophytin a; Pheide a—pheophorbide a; PE—phycoerythrin; C-PC—C-phycocyanin; PBPs—phycobiliproteins; Lyco—lycopene; Lo—loroxanthin; Neo—neoxanthin; Ax—antheraxanthin; Viola—violaxanthin; Ech—echinenone; Diato—diatoxanthin; Diadino—diadinoxanthin; DHA—docosahexaenoic acid; EPA—eicosapentaenoic acid; /—no data to our knowledge. * The classification of *Shizochytrium* sp. as microalga is controversial [40].

### 1.2. Probiotics as Health Supporters

The term probiotic, meaning “for life”, comes from the Greek language. Probiotics were first described in 1965 as “substances secreted by one organism which stimulate the growth of another organism” [89]. Today, probiotics are defined as “live microorganisms that, when administered in adequate amounts, confer a health benefit on the host” [90].

Gram-positive bacteria that belong to the genus Lactobacillus and Bifidobacterium are most commonly used as probiotics. In addition, *Lactococcus* spp., *Streptococcus thermophiles*, *E. coli* Nissle 1917, and yeast *Saccharomyces boulardii* are also used in the probiotic industry [91]. Probiotics have a variety of beneficial effects in humans and animals, including improving gut health, boosting immune response, lowering serum cholesterol levels, and preventing cancer. In recent years, probiotics have been increasingly recommended by medical professionals, especially gastroenterologists, as an effective therapeutic intervention [92,93]. Humans with certain diseases or health problems have a microbiota (symbiotic microbial cells that mainly colonize the intestine) that is different from the microbiota of healthy individuals [94]. Probiotics are used as agents that influence the function of the intestinal ecosystem and improve nutritional status and health [95]. The beneficial effects of probiotics are achieved by various mechanisms. They can activate specific genes in the host organism and stimulate its immunological response [96]. Moreover, as a part of the gut-brain axis, they control gastrointestinal hormone secretion and brain function via bidirectional neuronal communication [97]. They can also attach to intestinal epithelial cells and reduce the adhesion of pathogens in the gastrointestinal tract [98]. Probiotics produce a variety of secondary metabolites, some of which have been associated with health-promoting properties [95]. Among the most important are B vitamins and bioactive peptides. In recent years, the use of probiotics has expanded beyond intestinal wellness [99]. One example is the exploration of probiotic microorganisms as additives for cosmetics. This market is growing rapidly followed by the development of new products that contain probiotics, such as facial cleansers, foundations, face masks, etc. The market for probiotic cosmetics is expected to grow by nearly 12% between 2020 and 2030 [100]. Certain probiotic strains have abilities to improve the epithelial and epidermal barrier function [100]. *S. thermophilus* increases ceramide production and improves skin hydration in healthy individuals [101]. In addition, *Lactobacillus* cultures can improve the efficacy of deodorants, lotions, or foot sprays [102]. Therapies based on probiotics for atopic dermatitis and eczema are intensively researched in dermatology [103]. For example, *Bifidobacterium longum* lysate reduces factors associated with inflammation [104]. Accordingly, acne and hair loss are new indications for oral or topical administration of probiotics [99,105,106]. Probiotics have gained extreme popularity in recent decades, and the number of studies addressing this topic is increasing almost exponentially (35616 results in the PubMed database with the search query probiotics, 5068 in 2021 alone, assessed 040121). Although probiotic organisms have shown benefits in terms of stimulating the host immune system and antimutagenic and anticarcinogenic activity, they are strain-, dose-, and viability-dependent. For example, in the production of probiotic fermented milk, the viability of probiotics is lost during the fermentation process and cold storage [107]. To exert their beneficial effects, a high viability of lactic acid bacteria in the intestine must be achieved and maintained (10^6^ CFU g^−1^) [108]. While some of the health benefits are well documented, others may require further research [109]. A critical opinion on this topic was recently expressed in the study of Suez et al. [110], in emphasizing the importance of a mechanism-based approach in the administration of probiotic organisms. In addition, the goal of therapy should be precisely determined and based on individual medical indications. Due to the increasing popularity of using probiotics and algae for human and animal well-being, new ideas have emerged to combine these organisms and study their mutual response. Most studies consider algae as prebiotics to enhance probiotics’ positive health effects. However, the extreme diversity of algal organisms and their ability to produce a plethora of metabolites is leading to new experimental designs that combine the two organisms to benefit from their synergistic interactions.

## 2. Lessons from Natural Co-Culture Systems

Co-cultures can be divided into self-organized communities and assembled co-cultures. Self-organized communities include natural communities that can be further enriched and are usually the result of natural selection. Symbiotic relationships can be considered as natural co-culture systems. These types of close biological interactions play an important role in any type of microbial community. They encompass a wide range of interactions that can be mutualistic, commensalistic, or parasitic. Mutualism involves positive interactions between different microbial species that enhance the overall viability of the partners involved and are based on the exchange of resources and services [111]. These systems are very complex in terms of the number of microbial species involved and their mutual intercellular interactions.

The term phycosphere has been used since 1972 to describe a zone extending outward for an indefinite distance from an algal cell or colony in which bacterial growth is stimulated by the extracellular products of the alga [112]. The area serves as a dynamic and fluid environment containing many different types of chemical fluxes. Among other things, it is filled with fixed organic carbon ready for consumption by the bacteria. The fixed carbon is released by the algae, and only specific strains of bacteria can survive in this region rich in organic compounds. The mutual functioning of microalgae and bacteria requires enormous metabolic activity associated with a complex signaling network supported by a fluid genetic machinery. Interactions between microalgae and bacteria include nutrient exchange, signaling, and gene transfer. Nutrient exchange is the most common and, to date, the most important pathway of communication [113]. Various methods of communication exist within the symbiotic environments of bacteria and algae, including interbacterial and inter-algal communication, and interkingdom signaling. Studying how these two groups of organisms communicate with each other can improve our understanding of their behavior within phycospheres [114].

Natural microalgal-bacterial consortia are well described in the marine environment. Bacterial strains contribute to microalgal health by remineralizing organic material and/or converting necessary elements into bioavailable forms. Croft et al. [115] reported that large number of algae take up vitamin B12, which is essential for their growth. In addition, about 25% of existing microalgae could be auxotrophic for vitamin B1, and 8% for vitamin B7 [116]. A model describing the interaction between microalgae and bacteria in the ocean was created using the bacterium *Rugeria pomeroyi* DSS-3 and the diatom *Thalassiosira pseudonana* CCMP1335 [117]. The mutualistic interaction showed that *R. pomeroyi* supplies vitamin B12 to *T. pseudonana*, which, in turn, secretes 2,3-dihydroxypropane-1-sulfonate into the medium that the bacteria can use as a carbon source. In addition, the importance of the micronutrient iron for the growth of microalgae in the oceans has been well studied [118]. *Roseobacter* and *Marinobacter* produce a siderophore vibrioferrin that binds Fe (III) and makes it available for microalgae to use in the photosynthetic process and in fixing inorganic carbon. Some of the fixed carbon is released back into the medium as organic molecules that can be used for bacterial growth. Nitrogen is also one of the elements involved in nutrient exchange between algae and bacteria. The bacterium *Azobacter vinelandii* is able to fixate nitrogen, which can be used as inorganic nitrogen for microalgal growth [119]. This can be exploited to reduce the cost of the nitrogen source in the culture medium for large-scale cultures. In another study, a bidirectional interaction was found between *Scenedesmus* sp. LX1 and naturally co-occurring bacterial strains. In the presence of microalgae, ten out of twenty bacterial strains were able to produce and secrete indoleacetic acid (IAA), which promoted the growth of *Scenedesmus* sp. LX1 [120]. Thus, microalgae cultured with specific growth-promoting bacteria could be a potential strategy for improving large-scale microalgae cultivation in an economical and environmentally friendly manner.

## 3. Microalgae-Bacteria Consortia in Biotechnology

The second type of microbial communities includes both synthetic and artificial co-cultures created by human intervention [111]. Over the years, great efforts have been undertaken by various researchers to understand natural consortia and transfer this knowledge to an artificial consortium for specific biotechnological purposes [121]. Most studies focus on microalgae-bacteria consortia target wastewater treatment and biofuel production to minimize the high cultivation costs of microalgae production while removing pollutants from wastewater [122,123]. Synergistic interactions between microalgae and bacteria enable faster removal of pollutants in the consortium than in monocultures [122]. These include removal of several toxic metals, and even dissolved methane, as well as degradation of organic pollutants and other toxic pesticides, such as dichloro-diphenyl-trichloroethane (DDT) and atrazine [124,125].

Another approach to exploit the enormous potential of algae in terms of eco-engineering is the idea of biorefinery, where the ability of microalgae to produce different metabolites and products through subsequent extraction steps without waste generation can be exploited [126,127]. The interactions between microalgae and bacteria in such refineries cover the bioenergy sector to improve the quality of biodiesel and bioethanol production by increasing the biomass, lipid content, and productivity of microalgae [128]. The production of H_2_ in these systems can be improved by enhancing starch accumulation [119], combining the production of second-generation biofuels from microalgae biomass with aerobic bacteria to produce biogas [129], and generating electricity by using light microbial solar/fuel cells [130]. Under the economically favorable biorefinery concept, biomasses derived from co-cultures of photoautotrophic microorganisms and bacteria are reused as biofertilizers in agriculture. Biofertilizers play an important role in the decomposition of organic matter, providing better nutrient availability for plants [131]. Co-cropping systems showed better resistance to plant diseases and higher productivity of vegetable crops, including beans, corn, onions, and romaine lettuce [132,133,134,135]. In addition, mutualistic and/or commensalistic interaction have paved the way for the creation of novel platforms for the production of bioplastics. Polyhydroxyalkanoate (PHA) molecules are biodegradable and sustainable coproducts useful for many applications currently covered by petroleum-based plastics [136]. Thus, microalgae-bacteria co-cultures are finding their way into various biotechnological applications, including waste treatment, as well as production of environmentally acceptable fuels, bioplastics, and various other compounds.

## 4. Methods in Biotechnological Co-Cultivation of Algae and Bacteria

Most of today’s biotechnology industry is focused on production in axenic systems, which has its advantages in ease of operation and maintenance. However, co-culture systems are an interesting solution that can improve productivity and synthesis of active compounds. Moreover, the risk of contamination in axenic cultures can be reduced [137]. Various co-cultivation methods were reviewed by Kapoore et al. [138].

### 4.1. Experimental Setup for the Co-Culture of Algae and Bacteria

The inoculation ratio of bacteria to algae is one of the most important factors in co-cultivation when it comes to ensuring that both microorganisms are viable and not overpopulated. Usually, bacteria have faster growth rates than microalgae, so cell numbers of both microbes should be carefully optimized. Most of the time, this ratio benefits the microalgae because of their lower growth rate. As it is shown in Table 2, different inoculation ratios and growth conditions have been used in different studies. This is not surprising given the divergence of algae. In addition, downstream processing and analysis, as well as the ultimate goal of the study, should be considered when designing experiments.

**Table 2:** An overview of recent studies in the development of microalgae and probiotics co-cultivation. The table shows process parameters and inoculation ratios, as well as the aims of studies from different research studies.

Microalgae	Probiotic Microorganism	T [°C]	pH	Inoculum Ratio	Agitation	Aim of Study	Ref.
*Isochrysis* *galbana*	*Carnobacterium piscicola* *Lactobacillus brevis* *Lactobacillus casei* ssp. *casei*, *Lactobacillus helveticus* *Lactococcus lactis* spp. *Lactis* *Leuconostoc mesenteroides* spp. *mesenteroides* *Pediococcus acidilactici*	22 ± 1	No data	No data	Manually shaken twice daily	Effect on the growth rate of microalgae	[139]
*Chlorella* *sorokiniana*	*Bifidobacterium longum* *Lactobacillus plantarum*	4	No data	1:1000 1:1	Without	Evaluate microbial effects (antiviral) on rotavirus	[108]
*Nannochloropsis oceanica*	“Probiotic” bacterial strain isolates	25	8.5	6:1 30:1 60:1	100 rpm on an orbital shaker	Enhancing eicosapentaenoic acid (EPA) production	[140]
*Botryococcus braunii*	*Rhizobium* sp.	20 25 ± 2	No data	No data	No data	Enhancing growth rate	[141]
*Isochrysis* *Galbana*, *Chaetoceros calcitrans*	*Bacillus licheniformis* *Bacillus subtilis*	28 ± 1	8	4.5:1	Air-flow	In vitro growth of co-cultured microalgae and bacteria and their effect on oyster *C. sikamae*	[142]
*Chlorella* *sorokiniana*	*Azospirillum brasilanse*	28	7.2	1:1	Air-flow (with CO_2_) and stir bar	Investigation of oxidative stress in microalgae	[143]
*Spirulina platensis*	*Lactobacillus casei* *Lactobacillus acidophilus* *Streptococcus thermophilus*	37	6.8 6.2	/	No data	Stimulation of Lactic Acid bacteria growth with spirulina powder and their antibacterial activity	[144]
*Arthrospira platensis*	*Lacticaseibacillus casei* *Lacticaseibacillus rhamnosus*	37	No data	No data	Without	Evaluation of solid-state fermentation of *A. platensis* on two species of lactic acid bacteria	[145]
*Spirulina platensis*	*Lactobacillus acidophilus*	42	Endpoint pH = 4.6–4.7	No data	No data	Formulation of probiotic yogurts enriched with Spirulina biomass	[146]
*Planktochlorella* sp.	*Lactobacillus rhamnosus*	37	No data	/	No data	Prebiotic effect of algal extracts on growth of probiotic species	[147]

**Figure 2:**
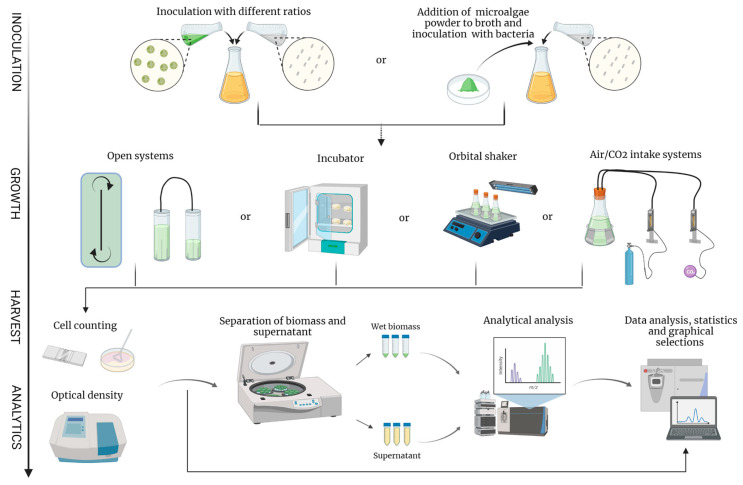
A contractive overview of the methods used for studies of microalgae and probiotic consortia. Co-cultivation begins with inoculation of microalgae and bacteria. Either viable microalga or its powder/extract can serve as an inoculum. The system in which the microbes are inoculated can be closed or open system (flow-through ponds; tubular photobioreactors); Petri dishes/biofilms in incubators; orbital shakers; or flasks with air/CO_2_ supply. During cultivation, growth parameters are usually measured by cell counting (CFU; hematocytometer) and optical density. Downstream processing is performed by separating biomass and supernatant, which are subjected to analytical analysis.

In a study by Kim et al. [148], porous microplates were used for co-culture of microalgae and bacteria. The temporal and spatial interaction between algae and algae-associated bacteria appears to be taxonomically dependent. While the diatom *Phaeodactylum tricornutum* had better biomass yield, the bacteria responded to the supply of inorganic and organic nutrients by the algae in a spatially predictable manner. Therefore, depending on the objectives of the study, precise growth conditions should be established for each cultivation pair to benefit from each organism. The most basic and commonly used method for culturing microalgae and bacteria, in general, is growth in a communal liquid medium (CLM) (Figure 2). This method includes direct mixing, pelleting, flocculation, or various types of biofilm formation as a mode of contact between species. Direct mixing is the most frequently used system for co-cultivation (Table 2). The effect of biofilm formation on the yield and productivity of microalgae in a co-culture was evaluated by Rivas et al. [57]. In this study, *Rhizobium* sp. acted as a probiotic and improved the performance of the microalga *Botryococcus braunii*. These results could be applied to large-scale cultivation, especially for microalgae with lower growth rates.

Other settings could also be easily adapted to co-cultivation systems. For example, gas exchange systems contain two vessels, in which one of the vessels is adapted to support autotrophic growth and the other heterotrophic. Santos et al. [149] improved both cultivation yield and lipid productivity using this system with a microalga that can act both as autotroph and heterotroph. The heterotrophic vessel from this research could easily be replaced in a co-culture system with heterotrophic bacterium that also produces carbon dioxide.

### 4.2. Downstream Processing and Analysis of Algae and Bacteria Co-Culture

After cultivation, biomass is usually harvested by various types of ultrafiltration steps, centrifugation, and electrocoagulation, which are not economically advantageous [108,139,140,142,143,146]. Pelletization and flocculation are usually used as low-cost downstream processing methods that reduce the cost of separating microalgae from the media. The process occurs when bacteria are added into the media, which, in turn, causes the microalgal cells to clump together and settle [6]. Recently, non-toxic bioflocculation is has been gaining more and more attention among researchers, to improve the harvesting of microalgal biomass. Bioflocculants play an important role in the process of flocculation and are composed of exopolymeric substances, exopolysaccharides (EPS), generally produced by bacteria and other microorganisms (yeasts and fungi) [150]. In addition to harvesting, another technique used in downstream processing is the disruption of microalgal cells to extract and refine the desired compound. Induced autolysis of microalgae is considered as a suitable method to replace the use of enzymes that cause the degradation of intracellular material. In this process, bacteria mainly attack and kill the targeted microalgae by releasing extracellular compounds [151]. Some algicidal molecules involved in the interaction process between bacteria and microalgae have been identified and include derivatives of alkaloids, pyrroles, quinolones, and enzymes. The degradation of microalgal cells using algicidal microorganisms in co-cultures is an effective digestion method, and even provides an excellent basis for further processing, such as biogas production or fermentation [125,152]. Therefore, a cost-effective digestion method needs to be developed to minimize the high production costs of the desired products. The focus should be on sustainability and ease of adaptation, without losing sight of the economic factor of the whole process [153].

In recent decades, the development of multi-omics approaches has enabled a deeper understanding of interactions between different species, both in natural and artificial co-cultures. Gene expression variations that reveal the physiology of individual microorganisms and their microbial responses in consortia can be assessed through transcriptomic studies [154,155]. Microarray hybridization, quantitative real-time polymerase chain reaction (qRT-PCR), and RNA-seq technology are primarily used for this type of analysis. Zhou et al. [156] performed transcriptomic analyses to understand the influence of the quorum-sensing molecule N-acyl-homoserine lactone (AHL) from mud bacteria on the expression of algal enzymes. Transcriptomic studies revealed upregulation of genes involved in vitamin B12 metabolism in algae in several studies and linked this metabolic switch to the response to co-cultivation with bacteria capable of producing this vitamin themselves [117,157]. The proteomic approach can provide in-depth insights into protein changes, such as post-translational modifications in response to environmental stimuli, most commonly through LS-MS (liquid chromatography coupled with mass spectrometry) and similar methods. In addition, analysis of the “secretome” can provide specific information on protein dynamics outside the cells [158]. A combination of transcriptomic and proteomic analysis of lipid production pathways enabled a major step toward the industrial application of lipid production by microalgae. These analyses provided detailed information on the regulation of lipid production in *C. vulgaris* under nitrogen-depleted conditions and a better understanding of enzyme activities and their biochemical functions in microalgal-bacterial consortia [159]. Proteomic tools also cover the up- and down-regulation of proteins useful for the ammonia oxidation pathway of bacteria under environmental stress conditions during wastewater treatment [160]. Transcriptome analysis can also be coupled with other omics approaches, such as metabolome identification, to link global changes in gene expression to the response of end cells to specific growth conditions or various environmental stresses. Durham et al. [117] identified changes in transcription products during development of consortia comprising microalga *Thalassiosira pseudonana* and bacteria *Ruegeria pomeroyi*. In addition, transcriptional responses were coupled with metabolome analyses to identify biogeochemically relevant candidate metabolites. Metabolomes are currently under investigation because they can be affected by environmental stress, genetic changes, and the physiological state of organisms [161]. It is necessary to understand metabolic interactions in microalgal-bacterial consortia to successfully alter partners. However, there are very few studies that focus on the metabolism of algae and their consortia, including bacterial strains. Changes in the nutrient composition of the culture medium directly affect the productivity of microalgae and bacteria. Therefore, these variations must be considered when optimizing yields of products of industrial interest, such as biofuels, pharmaceuticals, and pigments. These products of consortia biosynthesis can be detected by various spectrometric methods. Both Raman spectrometry and infrared spectrometry yielded distinct bands for different molecular arrangements in a quantitative study of metabolites [162]. The spectroscopic technique of Fourier transform infrared spectroscopy (FTIR) is also used for quantitative and qualitative analysis of a wide range of metabolites, such as carbohydrates, proteins, and nucleic acids, to identify bacteria and microalgae in the natural population and to detect changes under different stress conditions [163]. For example, a higher ratio of lipids to proteins after ATR (attenuated total reflectance)-FTIR has been observed in a mixotrophic consortia of *Phacus* sp., *Euglena* sp., *Phormidium* sp., *Chlorella* sp., and *Chlorococcum* sp. [164]. NMR (nuclear magnetic resonance) and MS (mass spectrometry) are the most promising technologies for metabolomic studies due to their high reproducibility. Although MS has higher sensitivity compared to NMR, NMR has recently gained attention because of easier sample preparation and high throughput in detecting primary and secondary metabolites. Metabolomics based on gas chromatography and MS have also contributed significantly to the detailed detection of consortia interactions. A study by Paul et al. [165] showed the influence of the bacterium *Dinoroseobacter shibae* on the metabolites of the phytoplankton *T. pseudonana*. The upregulation of amino acids and their derivatives was detected only when *D. shibae* was co-cultured with *T. pseudonana*. The use of metabolomics in consortia also provides insights into the composition of some unsaturated fatty acids that have high economic value [166]. Liquid chromatography (LC), coupled with quadrupole time-of-flight mass spectrometry (QTOF-MS), has been used for lipid profiling of microalgae [167,168]. By using LC in conjunction with mass analysis by Fourier transform ion cyclotron resonance mass spectrometry (FT—ICR-MS), the elemental structure of polar lipids in the green alga *Nannochloropsis oculata* was determined [169]. Higgins et al. [170] enabled understanding of bacterial cofactors that enhance algal metabolic capabilities by coupling ultra-particulate liquid chromatography (UPLC) with QTOF. Overall, a combination of all omics, including genomics, transcriptomics, proteomics, and metabolomics, plays an important role in understanding the interactions between microalgae and bacteria in a consortium.

## 5. Microalgae and Bacteria Consortia for Nutraceuticals

The general increase in health consciousness and preference for natural ingredients and flavors have led the food industry to shift toward the consumption of natural foods with health benefits, such as probiotics. On the other hand, microalgae are widely recognized as valuable foods and dietary supplements due to their excellent nutritional composition. An overview of some studies investigating the co-culture of microalgae and probiotic microorganisms is provided in Table 2.

**Figure 3:**
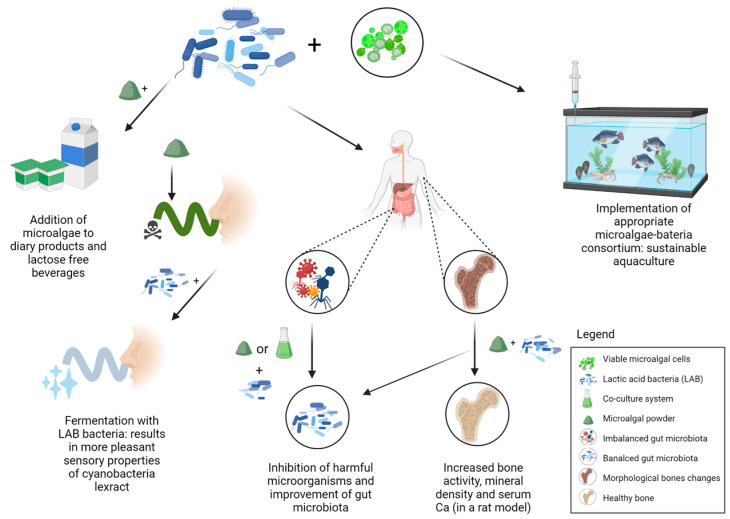
An overview of the implementation of microalgae and probiotic bacteria in co-culture systems. Dry microalgal powder combined with probiotic bacteria finds its application in various food additives approved for human consumption. Its synergistic action has been shown to improve the qualitative composition and sensory properties of various dairy products. It also has an inhibitory effect on viruses or bacteria that disturb the balance of the human intestinal microbiota and can potentially contribute to the bone health. Food preparations consisting of viable cells of microalgae in co-culture with probiotic bacteria are already finding application in sustainable aquaculture.

Most of the research focuses on microorganisms and algae that are used as biomass beneficial to bacteria (Figure 3). Often, microalgae are added to probiotic bacteria in the form of powder or extracts to improve bacterial growth and composition [144,145,146,147]. *A. platensis* is the most studied photosynthetic cyanobacteria in terms of dry biomass usage in food additives. Its dry biomass has been extensively studied to determine its effect on the growth of various lactic acid strains, such as *Lactobacillus* and *Bifidobacterium*. Bhomwik et al. [144] studied the effect of dry biomass of *A. platensis* on three lactic acid bacteria. The growth of *L. casei* MTCC 1423, *L. acidophilus* MTCC 447, and *S. thermophilus* MTCC 1938 was stimulated by the dry biomass of algal products derived from the late log phase of growth. In addition, the inhibitory effect on human pathogenic strains was observed in this study. *A. platensis* F&M-C256 biomass was also evaluated as a substrate for lactic acid fermentation by the probiotic bacterium *L. plantarum* ATCC 8014 to investigate the suitability of microalgal biomass in vegetable soy beverage or water. *A. platensis* biomass is reported to be a suitable substrate for *L. plantarum* 8014 growth, highlighting the potential of *A. platensis* biomass as a substrate for the production of new functional lactose-free beverages [171]. Patel et al. [146] succeeded in developing a probiotic Spirulina yogurt (PSY) rich in carotenoids without using food additives, such as stabilizers and acidity regulators, and *L. acidophilus* had excellent growth during fermentation in PSY and maintained its viability during storage. Another study analyzed the changes in flavor profiles after lactic acid fermentation of *A. platensis* biomass [145]. Before fermentation with *L. casei* 2240 and *L. rhamnosus* GG, two different stabilization treatments were performed: UV light treatment and sterilization. In addition to the suitability of the biomass as a matrix for solid-state fermentation, the fermentation process was also useful in reducing off-flavors. The odor and taste of microalgae can be unpleasant from the consumer’s point of view [146]. Heat treatment was found to be the most successful stabilization treatment as it resulted in improved aroma after fermentation. Thus, fermentation with lactic acid bacteria can be an interesting tool to obtain cyanobacterial biomass with more pleasant sensory properties for potential use in food formulations [145].

The unicellular microalga *Planktochlorella nurekis* produces bioactive compounds using the photoreprogramming method of metabolism [147]. Its biomass serves as a growth modulator of microorganisms used in the pharmaceutical and cosmetic industries. Treatment with the combination of red and blue light results in microalgal biomass with unique biochemical profiles, especially fatty acid composition. Ethanolic and aqueous extracts of algal biomass inhibited the growth of several pathogenic bacteria, such as *Pseudomonas aeruginosa*, *Escherichia coli* PCM 2209, and *Candida albicans* ATCC 14053, making them suitable for probiotic use. In addition, *P. nurekis* extracts had a prebiotic effect on the growth of *L. rhamnosus* ATCC 53103. These results indicate that oligo- and polysaccharides extracted from algae may provide an alternative source of prebiotics that can stimulate the growth of the probiotic bacteria *Lactobacillus* and *Bifidobacterium* [147]. Macroalgae are of particular interest as prebiotics due to their richness in polysaccharides [172]. Very recently, the role of algae as a prebiotic was reviewed by Patel et al. [173].

In vivo study by Hua et al. [174], using rats fed with a low calcium diet, tested *Chlorella pyrenoidosa* protein hydrolysate and calcium chelate (CPPH-Ca) on the intestinal and bone health. These treatments resulted in improved calcium absorption, bone activity, mineral density, and content, while inhibiting bone morphological changes and decreasing serum alkaline phosphatase. Moreover, the composition of microbiota was shifted toward probiotic bacteria, such as *Lactobacillus* and *Bifidobacterium*. Thus, intestinal microbiota may play an important role in attenuating metabolic abnormalities as a positive response to the algal CPPH-Ca supplement.

Recently, the co-cultivation of viable microalgae with bacteria has been gaining interest because the metabolic interactions of both organisms go beyond the role of algae as prebiotics (Figure 3). These can impart additional properties to the final product. In a study that examined *Isochrysis galbana* in co-culture with various probiotic microorganisms, this microalga was found to favor growth with bacteria, as evidenced by higher population numbers of the species [139]. In contrast, Sanchez-Ortiz et al. [142] reported no growth-promoting effect of *I. galbana* on other bacteria. Moreover, *B. subtilis* suppressed the growth of *I. galbana*. However, the cultivation parameters in the experiments were different (Table 2). Biocarriers are another way of co-culture implementation. *Chlorella vulgaris* was tested as a mechanical carrier of *Bacillus casei*, which resulted in a strong relationship and attachment of the bacteria to the surface of *C. vulgaris* [175]. This bioencapsulation suggests that it is possible to create a complex of algae and probiotic bacteria, together, as a feed supplement that enhances the function of the two already beneficial microorganisms.

Co-culture of algae and probiotics may have additional benefits besides growth enhancement. In a study by Cantú-Bernal et al. [108], the activity of *Chlorella sorokiniana* on the viability of *Bifidobacterium longum* and *Lactobacillus plantarum* in a dairy product and its microbial activity against rotavirus were evaluated. The results showed that *C. sorokiniana* not only significantly improved the viability of *L. plantarum* and *B. longum* in a dairy product but also increased their antiviral activity. This suggests that *C. sorokiniana* could be used as an ingredient for the development of products with additional health benefits [108].

## 6. Overview of Algae-Probiotics Co-Culture in Aquaculture

Until recently, very little attention was paid to bacterial strains in aquaculture. Their presence was usually associated with the control of bacterial diseases. Several algal species have also been used to control pathogenic bacteria in aquaculture systems by disrupting quorum sensing communication between pathogenic bacteria [176]. Selection of the appropriate consortium can significantly affect aquaculture production and sustainability (Figure 3). It was shown that consortia of algae and bacteria lead to healthier *Artemia* sp. cultures through better nitrogen assimilation [177]. Several studies have looked at treating aquaculture effluent with algae and bacteria and using the harvested biomass as feed for Pacific white shrimp, *Litopenaeus vannamei*, in the context of sustainable aquaculture [178,179]. Souza et al. [180] studied the gut microbiota of *Nile tilapia* fed the protist *Schizochytrium* sp. Male *tilapia* were fed a diet supplement containing 1.2% *Schizochytrium* sp., and changes were assessed after 105 days. Using next-generation sequencing, a greater number of bacteria from the Firmicutes group was detected in the algae-fed males compared to the control fish. Thus, the microalgae had modulatory effects on the gut microbiota, without affecting the structure of the intestinal villi [180]. The mollusk *Haliotis rufescens*, the red abalone, is an important aquaculture species, especially in North America. Mussels of various sizes were fed a natural diet of the macroalga *Macrocystis integrifolia* supplemented with a mixture of three bacterial species: *Vibrio* sp. C21-UMA, *Agarivorans albus* F1-UMA, and *Vibrio* sp. F15-UMA. After a period of 210 days, there was a significant increase in average survival and monthly growth compared to the non-supplemented control [181]. Improving nutrition in intensive aquaculture production systems is necessary to reduce stress, make optimal use of nutrients, and check the genetic potential of fish. In addition, well-chosen consortia of microalgae and bacteria can also mean better settlement of shellfish larvae. The current lack of knowledge leads to several major challenges. Therefore, it is important to gain deeper insight into the diversity and potential of bacteria-algal interaction mechanisms and understanding of the chemistry behind them.

## 7. Conclusions

Both algae and probiotic organisms are of great value for the well-being of humans and animals, as they produce various valuable compounds and have many beneficial effects on health. Microalgae-bacteria consortia are mostly studied for other biotechnological applications, such as wastewater treatment, biorefinery, and biofertilization. In food and nutrition biotechnology, algae are usually used as extracts that can improve probiotic performance. Recently, cultivation of live microalgae with probiotics has gained more attention because interspecies interactions can add more value to the final product. The growth improvement and production of bioactive compounds in both organisms, as well as activities against pathogens and positive effects on the consumer microbiota, can be obtained from such co-culture systems. Synergistic effects are evident when these two types of organisms are used in different aquaculture systems. Further studies are needed to confirm similar effects in humans. The demand for healthier foods and more natural products will likely drive this research in the future.
